# What is it like living with X-linked hypophosphatemia?: results from an Australian consumer survey

**DOI:** 10.1093/jbmrpl/ziaf027

**Published:** 2025-12-06

**Authors:** Jessica L Sandy, Naomi Ford, Sandy Bevc, Christine Rodda, Aris Siafarikas, Peter J Simm, Lucy Collins, Christie-Lee Wall, Andrew Biggin, Craig F Munns

**Affiliations:** Institute of Endocrinology and Diabetes, The Children's Hospital at Westmead, Westmead, NSW 2145, Australia; Faculty of Medicine and Health, Children’s Hospital Westmead Clinical School, University of Sydney, Westmead, NSW, 2145, Australia; XLH Australia, Australia; XLH Australia, Australia; Western Clinical School, University of Melbourne, Sunshine Hospital, St Albans, 3021, Victoria, Australia; Melbourne Medical School, University of Melbourne, Parkville, Victoria, Australia; Kids Research Institute Australia, Nedlands, WA 6009, Australia; Department of Endocrinology and Diabetes, Perth Children’s Hospital, Nedlands, WA, 6009, Australia; Institute for Health Research, University of Notre Dame, Fremantle, WA, 6160, Australia; Medical School, Paediatrics, University of Western Australia, Perth, WA, 6009, Australia; Centre for Hormone Research, Murdoch Children’s Research Institute, Melbourne, VIC, Australia; Department of Paediatrics, University of Melbourne, Melbourne, VIC, 3010, Australia; Department of Endocrinology and Diabetes, Royal Children’s Hospital, Melbourne, VIC, 3052, Australia; Department of Endocrinology and Diabetes, Royal Children’s Hospital, Melbourne, VIC, 3052, Australia; Institute of Endocrinology and Diabetes, The Children's Hospital at Westmead, Westmead, NSW 2145, Australia; Institute of Endocrinology and Diabetes, The Children's Hospital at Westmead, Westmead, NSW 2145, Australia; Faculty of Medicine and Health, Children’s Hospital Westmead Clinical School, University of Sydney%, Westmead, NSW, 2145, Australia; Faculty of Medicine, Child Health Research Centre, The University of Queensland, Brisbane, QLD, 4072, Australia; Department of Endocrinology and Diabetes, Queensland Children’s Hospital, Brisbane, QLD, 4101, Australia

**Keywords:** X-linked hypophosphatemia, rickets, osteomalacia, disability, psychology, quality of life

## Abstract

X-linked hypophosphatemia (XLH) is a rare, X-linked dominant condition with a high burden of both physical and psychosocial disease. This study aimed to describe the experience and burden of disease for children and adults living with XLH in Australia by inviting affected individuals and their carers to complete an online questionnaire. Of the 46 responses, half were completed by a person with XLH, and half by carers. Thirty percent were male, 33% were aged less than 18 yr. Median age at diagnosis was 2 yr (IQR 1.5-3.0). There was a high burden of surgical intervention: 59% reported 5 or more surgeries and 33% (15) had major dental procedures. Bone deformities and painful bones/joints were rated as the most impactful symptoms for both children and adults with XLH with 54% of participants rating emotional and physical burden of disease as equally impactful. Participants reported experiencing many psychosocial and financial challenges, including mental health disorders (including depression, anxiety, suicidal ideation, and self-harm), discrimination, and social isolation. Seventy percent reported it was difficult or very difficult living with XLH. Over 80% percent strongly agreed that lack of XLH awareness impacts on support services and health funding and agreed or strongly agreed that it is hard living with a condition that most Australians have never heard of. X-linked hypophosphatemia imparts a high physical, emotional, psychosocial, and mental toll on affected individuals. While the most impactful reported symptoms were musculoskeletal features, this survey emphasizes the degree of social and psychological challenges that individuals with XLH face. Some of these difficulties appear to be worsened by a lack of awareness. Patient advocacy and improving knowledge of rare diseases such as XLH is a key role of health professionals and should improve overall experience for affected individuals.

## Graphical Abstract

**Figure:**
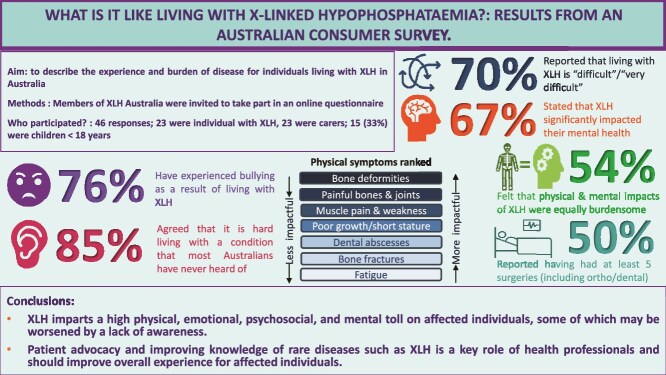

## Introduction

X-linked hypophosphatemia (XLH) is a rare, X-linked dominantly inherited lifelong and systemic disease. Clinical features are variable across the lifespan, and include rickets, osteomalacia, lower limb bowing, muscle and joint pain, fatigue, pseudo-fractures, craniosynostosis, deafness, and dental abscesses.^1–4^ The negative psychological and quality of life impact of XLH is being increasingly recognized but is still underappreciated.^4–9^ While it is the most common inherited form of rickets, XLH is a rare disorder, with the Australian estimated minimum prevalence at 1.33-1.91 per 10 000.^1^ XLH most commonly results from a de novo mutation in the *PHEX* gene; however, in subsequent generations for affected males, 100% of their female offspring will be affected and for affected females, 50% of their offspring (male and female) will be affected.^10^

In XLH, *PHEX* gene mutation results in an excess of FGF23 leading to renal phosphate wasting and subsequent hypophosphatemia.^2^ Treatment has traditionally been with oral phosphate and active vitamin D (calcitriol or alfacalcidol). This treatment is onerous and has significant side effects, such as an unpleasant taste of the phosphate supplementation and diarrhea, commonly limiting adherence.^8^^,^^11^^,^^12^ Conventional therapy has also been shown to be only somewhat effective in improving many of the clinical symptoms of XLH. Furthermore, inappropriate monitoring of treatment based on normalizing serum phosphate alone can lead to hyperparathyroidism, renal calculi nephrocalcinosis, and renal failure.^11^^,^^13^^,^^14^

Burosumab, a monoclonal antibody against FGF23, has recently been licensed and funded for XLH in many countries around the world, including Australia. This medication has been shown to be effective in both children and adults, normalizing serum phosphate levels, renal tubular resorption of phosphate, and ameliorating many of the clinical features of XLH, including rickets, pain, and limb deformity in children, and stiffness, pain, fracture healing, and mobility in adults.^15–18^

XLH Australia was established in 2018 as a patient support and advocacy group for people living with XLH.^19^ There are currently 200 members who are affected by XLH, of 1 which the majority reside in the states of New South Wales and Victoria. The survey was designed to better understand the impact of XLH on people’s lives. This study aimed to describe the experience and burden of disease for children and adults living with XLH in Australia in order to support raising awareness and improve patient advocacy.

## Methods

Individuals with XLH and their carers were invited to complete an online questionnaire (Appendix S1). The Checklist for Reporting Results of Internet E-Surveys (CHERRIES) checklist^20^ has been used to report the methods (see Appendix S3). This questionnaire included questions about demographics, clinical features, need for surgeries or dental procedures, and the physical and psychosocial burden of disease. The survey was initiated and designed by XLH Australia executive members, and conducted by Elbow Insight and Strategy, an independent market research agency. XLH Australia subscribers were invited to participate via social media (Facebook) and email and completed an online survey. This was an open survey promoted to cohort of approximately 200 members from XLH Australia. Completion of the survey was voluntary and there were no incentives offered. The survey was available to complete between December 13, 2021 and January 21, 2022. Participants were advised that the survey would take 15 min to complete. Participants were advised that all information collected in the survey would be kept confidential and results would be anonymized and aggregated into a report, in accordance with The Research Society’s Code of Conduct.^21^ Question numbers were not shown to participants of the survey; participants were shown a progress bar. Before proceeding to the main survey, participants were asked a few questions to determine if this survey was relevant to them. If participants selected that they worked for a telecommunications company, a marketing research company, or a pharmaceutical company, the survey concluded for those participants. Only those participants who selected they had been diagnosed with XLH or cared for a patient with XLH were able to continue to the survey. It is possible that a person with XLH who is also a carer for a child with XLH completed the survey twice. Respondents had to provide a response to move to the next question and could not review or change their responses once submitted.

Questions included open single response, numeric response, Likert scale, multiple response, open ended numeric, open ended, and rank order. For most multiple or single response questions, there was an option of “none of the above,” “unsure,” or “other” if they did not wish to choose a particular response. Multiple response or single response questions had randomization of response options to prevent bias. Adaptive questioning (certain questions conditionally displayed based on previous responses) was used to reduce number of unnecessary questions; the maximum number of items was 31. Multiple or single response questions with an option “Other” had an additional open-ended question for more information, the responses of which were then categorized and reported in a table format.

Data were collected prior to Australian Federal Government approvals by the Therapeutic Goods Administration (TGA) and Pharmaceutical Benefits Scheme (PBS) funding for burosumab.

Exemption of ethics review for this quality improvement project was sought and provided by Children’s Health Queensland Hospital and Health Service Human Research Ethics Committee, as the data that were collected and analyzed were un-identifiable and implied consent was obtained through completion of the survey. No personal information of participants was collected or stored.

Descriptive statistics were reported with mean ± SD for normally distributed data, or median (IQR) for nonparametric data. Differences between groups were analyzed for categorical variables using Chi square test and unpaired t test or ANOVA for continuous variables with significance taken at *p*-value <.05. Statistical analysis was done using IBM SPSS Statistics for Macintosh, Version 28.0.

Likert scale answers were represented visually in 100% stacked bar charts. When participants were asked to rank variables, weighted rank order analysis was performed to determine overall rank order. Open-ended questions were analyzed categorically with categories determined by a single investigator (JS).

## Results

Fourty-six responses were received, of which 50% (*n* = 23) were completed by individuals with XLH, and 23 were completed by their carers. Seventy percent of affected individuals reported on were female. Most carers (78%, *n* = 18) responded for someone less than 24 yr of age. Table 1 stratifies age, severity, and sex of affected individual. There were no incomplete survey responses.

**Table 1:** Age distribution and severity of disease of individuals with X-linked hypophosphatemia who responded and those whose carers responded.

**Age (yr)**	**Total (*n*)**	**Individuals with XLH % (*n*)**	**Carers % (*n*)**	**Mild % (*n*)**	**Moderate % (*n*)**	**Severe % (*n*)**
**<18**	15	0 (0)	100 (15)	20 (3)	53 (8)	27 (4)
**18-24**	5	40 (2)	60 (3)	20 (1)	40 (2)	40 (2)
**25-34**	4	100 (4)	0 (0)	0 (0)	25 (1)	75 (3)
**35-44**	8	63 (5)	38 (3)	25 (2)	63 (5)	13 (1)
**45-54**	8	88 (7)	13 (1)	0 (0)	75 (6)	25 (2)
**55-64**	3	67 (2)	33 (1)	0 (0)	33 (1)	67 (2)
**65-74**	2	100 (2)	0 (0)	0 (0)	100 (2)	0 (0)
**>75**	1	100 (1)	0 (0)	0 (0)	100 (1)	0 (0)

Abbreviation: XLH, X-linked hypophosphatemia.

Median (IQR) age at diagnosis was 2.0 yr (1.5-3.0). There was no significant difference in age at diagnosis between severity of illness categories; median (IQR) age was 2.0 yr (0.9-4.1), 3.0 yr (2.0-3.5), and 1.8 (1.3-2.0) yr in those reporting mild, moderate, and severe disease, respectively (*p* = .3). No affected individuals above the age of 44 yr categorized disease severity as mild. As seen in Table 2, there was a trend toward males reporting more severe disease, but this was not statistically significant (*p* = .56).

**Table 2:** Severity of disease based on sex.

**Severity of XLH**	**Total (*n*)**	**Total %**	**Male (*n*)**	**Male %**	**Female (*n*)**	**Female %**
**Mild**	6	13	0	0	6	19
**Moderate**	26	57	7	50	19	59
**Severe**	14	30	7	50	7	22

Abbreviation: XLH, X-linked hypophosphatemia.

As seen in Table 3, endocrinologists were the most likely health professionals to have made the diagnosis and be the primary care clinician followed by general practitioners. More children than adults were on treatment (87% vs 61%), but this was not a statistically significant result (*p* = .08).

**Table 3:** Clinician responsible for diagnosis and management of individuals with XLH.

	**% (*n*)**
**Clinician responsible for XLH diagnosis**	
**Endocrinologist**	50 (23)
**Nephrologist**	4 (2)
**Rheumatologist**	0 (0)
**General Practitioner**	11 (5)
**Pediatrician**	7 (3)
**Radiologist**	4 (2)
**Geneticist**	2 (1)
**Unsure/Other^a^**	20 (15)
**Primary managing clinician**	
**Endocrinologist**	83 (38)
**Nephrologist**	2 (1)
**Rheumatologist**	2 (1)
**General Practitioner**	35 (16)
**Other/Unsure^b^**	13 (6)

^a^“Other” = “specialist physician.”

^b^Other (free text option) = radiologist, no primary clinician, orthopedic surgeon, pediatrician, pediatric nurse, and multiple clinicians.

Abbreviation: XLH, X-linked hypophosphatemia.

As shown in Table 4, over half of the affected individuals had undergone 5 or more surgeries in the past, including osteotomies and dental extractions. Sixty-one percent (*n* = 28) had undergone at least 1 osteotomy, and 33% had had a major dental procedure. Mean ± SD age at first osteotomy was 13.4 ± 8.6 yr, and 23 ± 13 yr at most recent osteotomy. Of the 28 who had undergone an osteotomy, 32% (*n* = 9) took more than 6 mo to walk unassisted, 25% (*n* = 7) took 5-6 mo, 29% (*n* = 8) took 3-4 mo, and 11% took 1-2 mo. Thirty-three percent (*n* = 15) had major dental procedures, with a mean ± SD age of 14.5 ± 10.5 yr at first procedure and most recent tooth extracted at 30 ± 19 yr. Of those who had dental extractions, mean ± SD number of teeth removed was 3.9 ± 5.5 primary teeth and 6.1 ± 9.7 adult teeth.

**Table 4:** Surgical procedure burden in XLH.

	**Number of patients *n* = 46**	**Proportion %**
**Number of surgeries undergone**		
** None**	7	15
** 1**	1	2
** 2**	8	17
** 3**	3	7
** 4**	2	4
** 5 or more**	23	50
** Unsure**	2	4
**Number of osteotomies undergone**		
** None**	9	20
** 1**	1	2
** 2**	12	26
** 3**	7	15
** 4**	4	9
** 5 or more**	4	9
** Unsure/Unanswered**	9	20
**Number of dental procedures such as extractions**		
** None**	6	10
** 1**	5	14
** 2**	6	15
** 3**	5	6
** 4**	5	7
** 5 or more**	9	13
** Unsure/Unanswered**	10	12

Abbreviation: XLH, X-linked hypophosphatemia.

Table 5 shows weighted rank data analysis and demonstrates that bone deformities and painful bones/joints were the most impactful symptom reported for both children and adults with XLH. Fatigue and bone fractures were the least impactful symptom for both groups. Short stature/poor growth was rated as more impactful by children than adults, while adults ranked dental abscesses higher than children.

**Table 5:** Weighted rank data analysis of most impactful clinical feature.

	**Weighted rank data analysis**	**Rank**
**All individuals with XLH (*n* = 46)**		
**Bone deformities, such as knock knees or bowed legs**	251	1
**Painful bones and joints**	249	2
**Muscle pain and weakness**	191	3
**Poor growth/short stature**	179	4
**Dental abscesses**	157	5
**Bone fractures**	133	6
**Fatigue**	132	7
**Individual <18 yr of age (*n* = 15)**		
**Bone deformities, such as knock knees or bowed legs**	90	1
**Painful bones and joints**	78	2
**Poor growth/short stature**	73	3
**Muscle pain and weakness**	53	4
**Dental abscesses**	45	5
**Bone fractures**	42	6
**Fatigue**	39	7
**Individual >18 yr of age (*n* = 31)**		
**Painful bones and joints**	171	1
**Bone deformities, such as knock knees or bowed legs**	161	2
**Muscle pain and weakness**	138	3
**Dental abscesses**	112	4
**Poor growth/short stature**	106	5
**Fatigue**	93	6
**Bone fractures**	91	7

Abbreviation: XLH, X-linked hypophosphatemia.

Respondents were asked if the physical (pain, discomfort, and restricted mobility) or emotional/mental burden of disease were the greatest challenge. Over half (54%, *n* = 25) reported that the physical and emotional impacts were equally as burdensome, while 33% (*n* = 15) reported that physical aspects were the greatest challenges, and 11% (*n* = 5) stated that the emotional and mental burden were the greatest challenge.

**Figure 1:**
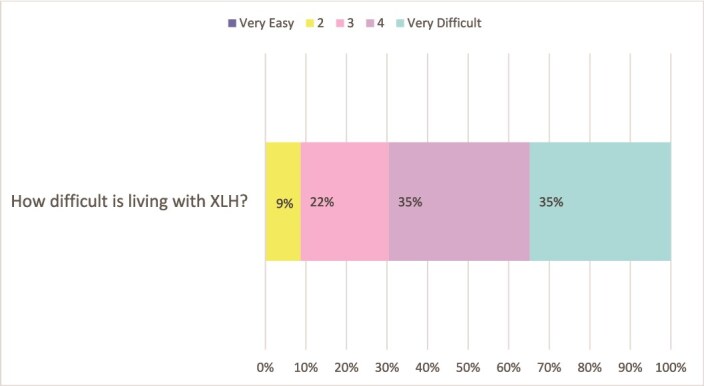
How difficult is living with X-linked hypophosphatemia: responses to Likert scale.

**Figure 2:**
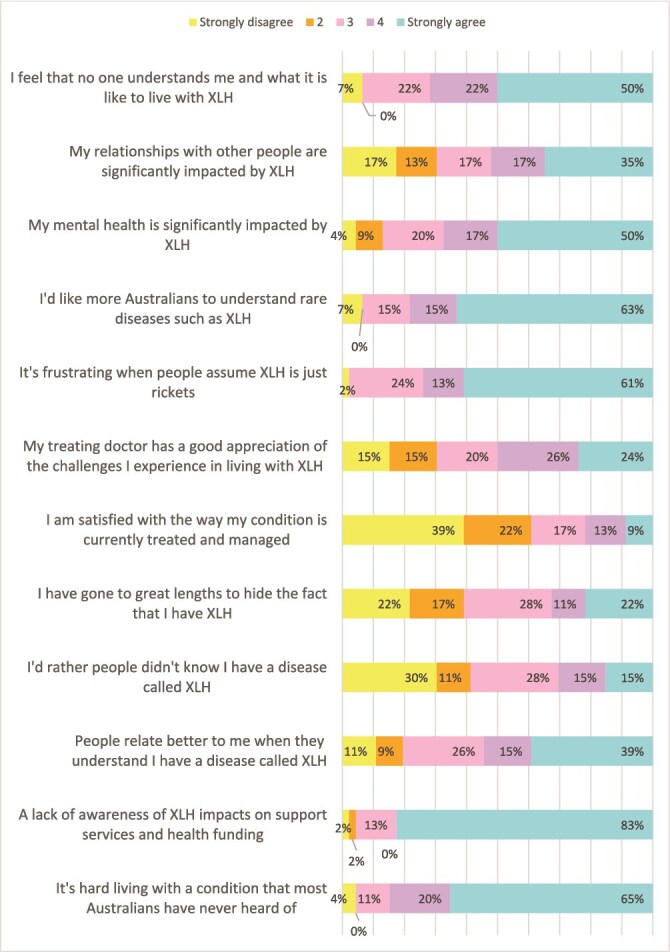
Responses to Likert scale questions on living with X-linked hypophosphatemia.

**Figure 3:**
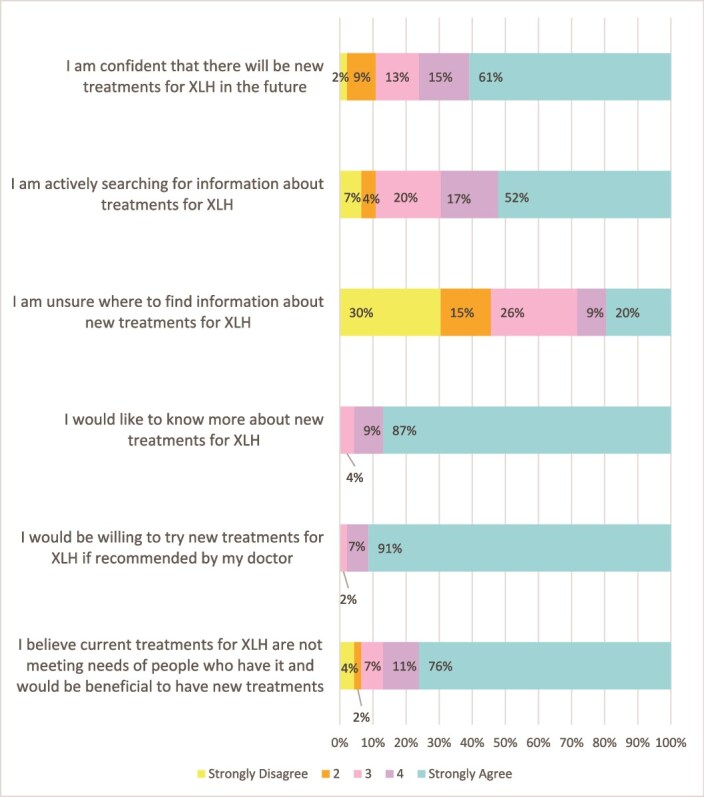
Responses to Likert scale questions on X-linked hypophosphatemia therapies.

Participants were asked questions with a Likert 5 scale response about living with XLH, which are summarized in Figures 1-3. Seventy percent (*n* = 32) reported that it was difficult or very difficult living with XLH. Figure 2 and Table 6 illustrate the extensive impact that XLH has on the lives of affected individuals, with a high proportion of respondents reporting impacts on relationships, daily life, and mental health. Most agreed or strongly agreed that it was hard living with a condition that most Australians were not familiar with, and that a lack of awareness also impacted on services and funding. Over half of respondents (54%, *n* = 25) felt that people related better to them when they understood that they had a disease called XLH. In contrast, approximately one-third of individuals indicated they preferred people not to know their diagnosis or that they had gone to great lengths to hide their disease.

**Table 6:** Proportion of individuals with X-linked hypophosphatemia who experience psychological or social difficulties.

	**Yes (*n*)**	**%**
**Have you experienced the following as a result of living with XLH?**		
**Bullying**	35	76
**Discrimination**	23	50
**Social isolation**	18	39
**Challenges relating to education**	15	33
**Difficulty securing or holding down a job**	11	24
**Difficulty making or keeping friends**	14	30
**Challenges relating to relationships**	18	39
**Difficulty making decisions about having children**	24	52
**Financial difficulties**	11	24
**Mental health challenges**	33	72
**Challenges with general activities of daily living**	39	85
**None of the above**	2	4
**Have you experienced the following at some stage?**		
**Low self-esteem**	38	83
**Clinical depression**	18	39
**Anxiety disorder**	29	63
**Self-harm**	10	22
**Suicidal thoughts**	11	24
**None of the above**	6	13

Abbreviation: XLH, X-linked hypophosphatemia.

As seen in Figure 3, most individuals with XLH were interested in knowing more about or trying new treatments for XLH and believed that current treatments, which at the time were oral phosphate and calcitriol therapy, were not meeting the needs of people with XLH.

**Figure 4:**
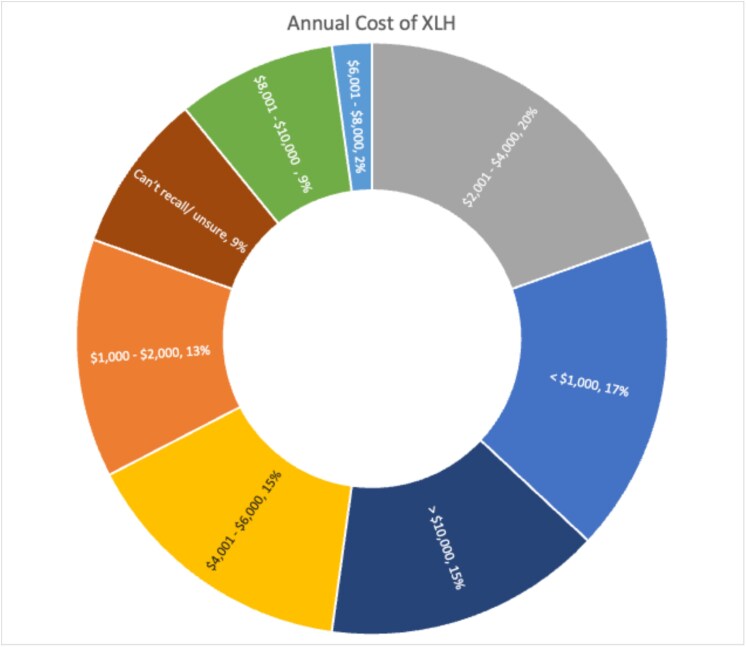
Annual cost of living with X-linked hypophosphatemia.

Estimated financial burden (displayed in Figure 4) was variable; approximate annual out of pocket costs associated with XLH were greater than $10 000 for 15% (*n* = 4), between $4000 and $10 000 for 26% (*n* = 12), and less than $4000 for 50%.

Participants were asked to comment on what they are hoping for in the future. All participants wrote a response. Responses are available in the supplementary appendices. Eighty-five percent (*n* = 39) expressed desire for approval and access to new treatments such as burosumab therapy. Twenty percent (*n* = 9) hoped for an increased awareness, 15% (*n* = 7) wished for resolution of physical symptoms, and 17% (*n* = 8) wanted to lead a more “normal” life and/or feel more included or integrated in society.

## Discussion and conclusions

This research highlights the significant burden of disease in XLH that spans across physical, psychological, social, and emotional domains, and persists throughout the lifespan. In particular, these data contribute to a growing, but still limited, body of evidence for the high psychological and social impact of disease in XLH. These results also highlight common frustrations of affected individuals around lack of awareness of clinicians and the community about XLH. Patient advocacy and improving knowledge of rare diseases such as XLH is a key role of the health professional and would be expected to improve overall experience for affected individuals.

X-linked hypophosphatemia has historically been thought of as mostly a disease of childhood. While it is recommended that symptomatic adults are treated,^22^ many individuals are not on any treatment despite experiencing pain or other symptoms, and often do not see a doctor for their XLH.^9^^,^^11^^,^^23^ This may reflect poor understanding of XLH by adult clinicians who may consider XLH as a condition confined to childhood, and furthermore may underscore the need for robust transition services for older adolescents with XLH to become engaged in adult healthcare. In addition, despite evidence of clinical improvement in clinical manifestations, including pain and function,^24^^,^^25^ burosumab is not available for adults with XLH in many countries. Our survey responses illustrate the significant burden of disease throughout the lifespan, and it is notable that all adults older than 44 yr of age reported their degree of illness was moderate or severe, compared with 19% of those younger than 44 yr reported having mild disease.

The high burden of orthopedic disease seen in our data supports previous literature.^1^^,^^9^ A recent systematic review revealed 57%-82% of adults reported previous orthopedic surgeries,^9^ and a recently published clinician survey reported that 42% of Australian and New Zealand children with XLH had undergone orthopedic intervention.^1^ Our study recorded a very high rate of orthopedic surgeries, with only 15% reporting no surgeries at all and 50% reporting 5 or more previous surgeries. There is likely a degree of selection bias in these data, as individuals (or carers of individuals) who are more impacted by XLH may be more engaged with advocacy groups such as XLH Australia and more motivated to participate in research questionnaires. However, these data do contribute to the body of evidence that orthopedic intervention imparts a significant impact on individuals with XLH. While it seems probable that the improvements in leg deformity and growth seen with burosumab therapy will lead to a reduction in surgical burden for affected individuals,^17^^,^^26–28^ there is need for future research in this area to provide more definitive evidence and to further justify the financial and overall benefits of government funding of such medications.

Dental manifestations, such as dental abscesses and requirement for dental extractions, impart a high burden of disease and impact quality of life for individuals with XLH.^29^^,^^30^ In this study, approximately one-third of affected individuals had undergone major dental procedures, with many requiring multiple tooth extractions. This is consistent with previous studies, including a recent Australian Pediatric Surveillance Unit clinician survey,^1^ which identified that 44% of children with XLH had a history of tooth abscess or extraction, with up to 5 abscesses per person at the time of reporting. Previous research has suggested that a lack of awareness of XLH impacts oral health–related quality of life,^30^ so involvement of specialized dentists within a multidisciplinary framework may help to streamline and improve the dental care of these individuals. The effect of long-term burosumab therapy on dental manifestations is uncertain, with existing literature showing conflicting results;^26^^,^^31^^,^^32^ future studies are needed to guide dentists managing these patients going forward.

While physical manifestations are important and were most often ranked the most impactful symptom for affected individuals in this study, the extent of psychological, social, and emotional burden of disease described in these data is substantial. These findings support previous literature highlighting the psychosocial impact of XLH^1^^,^^9^^,^^33^ and support a need to better integrate psychological care into management of individuals with XLH.^8^ These data highlight the considerably high rate of psychological or social difficulties seen in respondents, with over half reporting low self-esteem, bullying, discrimination, challenges with general activities of daily living, difficulties making decisions about having children, mental health challenges, and/or an anxiety disorder. While the impact on health-related quality of life and psychological well-being is being increasingly recognized in both pediatric and adult XLH, these data highlight focus areas for further research to better support individuals with XLH.^8^^,^^9^^,^^33^ Our data are consistent with previous literature that shows people with XLH have poorer health-related quality of life than unaffected individuals, with a greater impact seen in adulthood.^6^^,^^23^ This includes issues with mobility, self-care, self-esteem, bullying, the ability to do housework and/or work, and symptoms of isolation, anxiety, or depression.^9^^,^^33–35^ Clinical trials have documented improvement in pain and function with burosumab and there is some evidence that burosumab therapy improves quality of life,^36^^,^^37^ but there have been no large or long-term studies looking specifically at improvement in quality of life, or psychological, social, or emotional well-being with burosumab.^38^ Going forward, it will be important to better define the impact of novel therapies, such as burosumab, on quality of life and psychosocial well-being in XLH, especially in adulthood.

Most respondents did not wish to hide their illness and reported a wish for better education of clinicians and the community. This is consistent with the literature,^9^^,^^33^^,^^39^ particularly in the adult age groups, where, as discussed above, a lack of awareness of the need for treatments sometimes contributes to overall disease burden. Qualitative research has also highlighted a desire from individuals with XLH for improved awareness of their disease.^33^ Therefore, public health and targeted education programs to increase awareness of XLH and the potential impacts of this disease may improve the experience for affected individuals. In general, a lack of awareness leading to increased isolation, delayed diagnosis, and difficulties finding appropriate care is a common issue for all individuals with rare diseases.^40^ Recently, an increased appreciation for these challenges has led to a National Strategic Action Plan for Rare Diseases, an initiative launched by and the Australian government department of health in 2020 after advocacy by Rare Voices Australia and its stakeholders (including XLH Australia).^41^ This initiative focuses on 3 pillar—awareness and education, care and support, and research and data. Collaborative initiatives such as this are integral in maximizing progress in rare diseases, as the needs of many with rare diseases are similar to those with XLH. Going forward, ongoing collaboration and involvement in such programs, ensuring that the needs of those with XLH are being addressed by these initiatives, would be beneficial.

Conversely, it is important to note that around one-third of participants preferred that people did not know their diagnosis or that they had gone to great lengths to hide their disease, and many wished for “a normal life” or better integration and acceptance into society. To our knowledge, this has not been reported in previous research and may be useful to explore in more depth. In particular, future qualitative research taking into account the wider context and psychological well-being of affected individuals may provide valuable insights.

This research was completed prior to government approval and subsidization of burosumab in Australia. Most individuals indicated that they were dissatisfied with available treatments and expressed a desire for new treatments, especially burosumab, to be made available. Of relevance, this treatment is still not yet available in many countries, including New Zealand, and is not available for all age groups in others, for example, in some European countries where it is not available for affected adults. Burosumab has been shown in clinical trials to be effective in mitigating many of clinical features of XLH in both pediatric and adult age groups.^16^^,^^17^^,^^24–26^ Therefore, advocacy to government and funding bodies is needed for equal and equitable access to this important medication across the world.

Strengths of this study include the holistic nature of the questionnaire with consumer input into questions asked, as well as Likert scale and ranking style questions that effectively illustrate the overall burden of disease in XLH extending beyond the recognized physical symptoms. While the study size of 46 does not allow for a more in-depth statistical analysis, it represents a reasonable cohort for a rare disorder. Study design with recruitment via social media and email evidently introduces the possibility of selection bias. Studies have shown that those with poor mental health are less likely to participate in surveys,^42^^,^^43^ so these data possibly represent an underestimate of mental illness. In contrast, it is also likely that those who agree to participate are responding for individuals who are more impacted by XLH or have a particular motive for participating such as advocating for availability of new treatments such as burosumab. We saw a high burden of orthopedic disease in this cohort relative to recent literature, which indicates selection bias toward more severely affected individuals. It may have also been useful to collect data on treatment regimens and other demographics to compare the participants with baseline populations. This study was advertised to XLH Australia members and advertised on public social media and email. Individuals who are members and active participants in XLH Australia and social media are possibly less likely to report a desire for secrecy regarding their illness, and more likely to want others to know about it. The anonymity of the survey reduces reporting bias. Other limitations include the open survey design with no strategies to prevent individuals from completing the questionnaire more than once.

These data present important and comprehensive information that has not previously been described on the burden of disease beyond the physical symptoms of XLH in Australia. This study emphasizes the life-long and systemic nature of this condition, rather than it being solely a musculoskeletal disorder of childhood. This research highlights several important areas for further research or development. Future studies may look to better define the prevalence, predictors, and protective factors of mental health and psychosocial issues in XLH in Australia and other countries. Furthermore, development of an evidence-based and holistic approach to caring for individuals with XLH to reduce burden of psychosocial disease would be beneficial. Advocacy and improving knowledge of XLH in the medical and general communities may improve the experience of individuals with XLH. Longer term studies identifying the effect of long-term burosumab in XLH, including on psychosocial manifestations and quality of life, are important for advocacy and future planning.

## Supplementary Material

Supplemental_Appendix_1-Questionnaire_ziaf027

Supplemental_Appendix_2_ziaf027

CHERRIES_checklist_ziaf027

## Data Availability

The data underlying this article will be shared on reasonable request to the corresponding author.
