# Supplementary material for: What is it like living with X-linked hypophosphatemia?: results from an Australian consumer survey
Source: JBMR Plus. 2025 Dec 6;9(Suppl 5):v3–v13. doi: 10.1093/jbmrpl/ziaf027 (PMC12723801; doi:10.1093/jbmrpl/ziaf027)
Supplement: Supplemental_Appendix_1-Questionnaire_ziaf027 [file supplemental_appendix_1-questionnaire_ziaf027.docx]

# **Supplemental Appendix 1: XLH Australia Consumer Survey**

| **S1** | To start with, do you, or anyone in your household, work in any of the following?  SINGLE RESPONSE | | |
| --- | --- | --- | --- |
|  | A market research company |  | CLOSE |
|  | An advertising company |  | CLOSE |
|  | A pharmaceutical company |  | CLOSE |
|  | A telecommunications company |  | CONTINUE |
|  | None of these |  | CONTINUE |

| **S2** | Which of the following conditions have you been diagnosed with or confirmed to have by a **doctor**?  MULTIPLE RESPONSE; RANDOMISE | | |
| --- | --- | --- | --- |
|  | X-linked hypophosphataemia (XLH) | 1 | CONTINUE |
|  | Rheumatoid Arthritis | 2 | IF SELECTED ONLY CODES 2 TO 6, GO TO S2A |
|  | Cardiovascular Disease | 3 |  |
|  | Asthma | 4 |  |
|  | Diabetes | 5 |  |
|  | Other | 6 |  |
|  | None of the above | 7 |  |

| **S2a** | [IF S2 NOT EQUAL TO CODE 1] Do you care for someone who has the following conditions?  PROGRAMMER INSTRUCTIONS | | |
| --- | --- | --- | --- |
|  | X-linked hypophosphataemia (XLH) |  | CONTINUE  PROG: TAG AS ‘CARER’ |
|  | Rheumatoid Arthritis |  |  |
|  | Cardiovascular Disease |  |  |
|  | Asthma |  |  |
|  | Diabetes |  |  |
|  | Other |  |  |
|  | None of the above |  |  |

| **S3** | Are you…?  CARER: Please select the gender of the person with XLH that you care for, below.  SINGLE RESPONSE | | |
| --- | --- | --- | --- |
|  | Male |  |  |
|  | Female |  |  |
|  | Other |  |  |

| **S4** | Which age group do you fall into?  CARER: Which age group does the person with XLH that you care for fall into?  SINGLE RESPONSE \| DROP DOWN | | |
| --- | --- | --- | --- |
|  | Below 18 years old |  |  |
|  | 18 - 24 |  |  |
|  | 25 – 34 |  |  |
|  | 35 – 44 |  |  |
|  | 45 – 54 |  |  |
|  | 55 – 64 |  |  |
|  | 65 – 74 |  |  |
|  | 75 or older |  |  |
|  | Prefer not to say |  |  |

| **S5** | Which state/territory do you live in?  SINGLE RESPONSE | | |
| --- | --- | --- | --- |
|  | NSW | 1 |  |
|  | VIC | 2 |  |
|  | QLD | 3 |  |
|  | TAS | 4 |  |
|  | SA | 5 |  |
|  | WA | 6 |  |
|  | NT | 7 |  |
|  | ACT | 8 |  |

| **S6** | How would you classify the severity of [P: your XLH / C: the XLH of the person that you care for]? SINGLE RESPONSE | | |
| --- | --- | --- | --- |
|  | Mild | 1 |  |
|  | Moderate | 2 |  |
|  | Severe | 3 |  |
|  |  |  |  |

| **Q1** | [ALL] At what age [P: were you / C: was the person you care for] diagnosed with XLH?  NUMERIC | | |
| --- | --- | --- | --- |
|  | Years | ____________ |  |
|  | Months | [0-11 range] |  |
|  | Can’t recall/ unsure | ○ |  |

| **Q1a** | [ALL] [P] Please recall the time when you were diagnosed with XLH, which of the following type of doctors made the diagnosis (i.e. confirmed that you have the condition)?  [C] Please recall the time when the person you care for was diagnosed with XLH, which of the following type of doctors made the diagnosis (i.e. confirmed that you have the condition)?  SINGLE RESPONSE. RANDOMISE | | |
| --- | --- | --- | --- |
|  | Endocrinologist | 1 |  |
|  | Renal physician | 2 |  |
|  | Rheumatologist | 3 |  |
|  | GP | 4 |  |
|  | Other, specify _________ | 5 |  |
|  | Unsure/ can’t recall | 6 |  |

| **Q1b** | | [ALL] [P] Are you currently under the care of a doctor for XLH? If so, what type of doctor?  [C] Is the person you care for currently under the care of a doctor for XLH? If so, what type of doctor?  SINGLE RESPONSE. RANDOMISE | | | | | |
| --- | --- | --- | --- | --- | --- | --- | --- |
|  | | Endocrinologist | | 1 |  | | |
|  | | Renal physician | | 2 |  | | |
|  | | Rheumatologist | | 3 |  | | |
|  | | GP | | 4 |  | | |
|  | | Other, specify _________ | | 5 |  | | |
|  | | Unsure/ can’t recall | | 6 |  | | |
| **Q2** | [ALL] Overall, how would [P: you / C: the person you care for] describe living with XLH? Rate on a scale of 1 to 5 where 1=very easy and 5=very difficult.  SINGLE RESPONSE. | | | | | |  |
|  | Overall experience in living with XLH | | 1 2 3 4 5 | | |  |  |
|  |  | |  | | |  |  |

| **Q3** | [ALL] Based on [P: your experience / C: the experience of the person you care for] of XLH physical symptoms, rank the following in terms of impact where 1 is the MOST impactful and 6 is the LEAST impactful.  RANK ORDER. RANDOMISE | | |
| --- | --- | --- | --- |
|  | Bone deformities, such as knock knees or bowed legs | 1 |  |
|  | Painful bones and joints | 2 |  |
|  | Muscle pain and weakness | 3 |  |
|  | Bone fractures | 4 |  |
|  | Dental abscesses | 5 |  |
|  | Poor growth / short stature | 6 |  |
|  | Fatigue | 7 |  |
|  | None of the above | 8 |  |
|  |  |  |  |

| **Q4** | [ALL] Please indicate the extent to which [P: you agree or disagree / C: the person you care for agrees or disagrees] with the following statements. Rate on a scale of 1 to 5 where 1=strongly disagree and 5=strongly agree.  SINGLE RESPONSE PER ROW. RANDOMISE | | |
| --- | --- | --- | --- |
| 1 | It’s hard living with a condition that most Australians have never heard of  [C: The person I care for finds it hard living with a condition that most Australians have never heard of] | 1 2 3 4 5 |  |
| 2 | A lack of awareness of XLH impacts on support services and health funding  [C: The person I care feels that a lack of awareness of XLH impacts on support services and health funding] | 1 2 3 4 5 |  |
| 3 | People relate better to me when they understand I have a disease called XLH  [C: People relate better to the person I care for with XLH when they understand the disease called XLH] | 1 2 3 4 5 |  |
| 4 | I’d rather people didn’t know I have a disease called XLH  [C: The person I care for would rather people didn’t know that he/she has a disease called XLH] | 1 2 3 4 5 |  |
| 5 | I have gone to great lengths to hide the fact that I have XLH  [C: The person I care for has gone to great lengths to hide the fact he/she has XLH] | 1 2 3 4 5 |  |
| 6 | I am satisfied with the way my condition is currently treated and managed  [C: The person I care for is satisfied with the way their XLH is currently treated and managed] | 1 2 3 4 5 |  |
| 7 | My treating doctor has a good appreciation of the challenges I experience in living with XLH  [C: The treating doctor of the person I care for has a good appreciation of the challenges he/she experiences in living with XLH] | 1 2 3 4 5 |  |
| 8 | It’s frustrating when people assume XLH is “just rickets”  [C: The person I care for is frustrated when people assume XLH is “just rickets”] | 1 2 3 4 5 |  |
| 9 | I’d like more Australians to understand rare diseases such as XLH  [C: The person I care for would like more Australians to understand rare diseases such as XLH] | 1 2 3 4 5 |  |
| 10 | My mental health is significantly impacted by XLH  [C: The mental health of the person I care for is significantly impacted by XLH] | 1 2 3 4 5 |  |
| 11 | My relationships with other people are significantly impacted by XLH  [C: Relationships with others are significantly impacted by XLH for the person I care for | 1 2 3 4 5 |  |
| 12 | I feel that no one understands me and what it is like to live with XLH  [C: The person I care for feels no one understands him/her and what it is like to live with XLH] | 1 2 3 4 5 |  |
|  |  |  |  |

| **Q5** | [ALL] Thinking about living with XLH, which of the following statements best applies to [P: you / C: the person you care for]?  SINGLE RESPONSE. RANDOMISE CODES 1 & 2 | | |
| --- | --- | --- | --- |
|  | The pain, discomfort and restricted mobility are the greatest challenges | 1 |  |
|  | The emotional and mental burden are the greatest challenges | 2 |  |
|  | The physical and emotional impacts are equally burdensome | 3 |  |
|  | None of the above | 4 |  |

| **Q6** | [ALL] Please indicate which of the following [P: have you experienced / C: has the person you care for experienced] as a result of living with XLH? Select all that apply.  MULTIPLE RESPONSE. RANDOMISE | | |
| --- | --- | --- | --- |
|  | Bullying |  |  |
|  | Discrimination |  |  |
|  | Social isolation |  |  |
|  | Challenges relating to education |  |  |
|  | Difficulty securing or holding down a job |  |  |
|  | Difficulty making or keeping friends |  |  |
|  | Challenges relating to relationships |  |  |
|  | Difficulty making decisions about having children |  |  |
|  | Financial difficulties |  |  |
|  | Mental health challenges |  |  |
|  | Challenges with general activities of daily living |  |  |
|  | Other, specify ___________ |  |  |
|  | None of the above |  |  |

| **Q7** | [ALL] Thinking further about the impact of XLH, which of the following have [P: you / C: the person you care for] experienced at some stage during your life? Select all that apply.  MULTIPLE RESPONSE. RANDOMISE | | |
| --- | --- | --- | --- |
|  | Low self esteem |  |  |
|  | Clinical depression |  |  |
|  | Anxiety disorder |  |  |
|  | Self-harm |  |  |
|  | Suicidal thoughts |  |  |
|  | Other, specify ____ |  |  |
|  | None of the above |  |  |

**XLH Treatment**

| **Q8** | [ALL] Now thinking about the cost involved in living with XLH, approximately how much [P: do you spend / C: is spent on the person you care for] in an average year on medical appointments, treatments, equipment, and services relating to XLH?  Note: please estimate out-of-pocket costs i.e. cost that is not reimbursed by Medicare and NDIS.  NUMERIC | | |
| --- | --- | --- | --- |
|  | Less than $1,000 | 1 |  |
|  | $1,000 to $2,000 | 2 |  |
|  | $2,001 to $4,000 | 3 |  |
|  | $4,001 to $6,000 | 4 |  |
|  | $6,001 to $8,000 | 5 |  |
|  | $8,001 to $10,000 | 6 |  |
|  | More than $10,000 | 7 |  |
|  | Can’t recall/ unsure | ○ |  |

| **Q9** | [ALL] Are [P: you / C: the person you care for] currently receiving treatment for XLH?  SINGLE RESPONSE | | |
| --- | --- | --- | --- |
|  | Yes | 1 |  |
|  | No | 2 |  |
|  | Can’t recall/ unsure | 3 |  |

| **Q10** | [ALL] Are [P: you / C: the person you care for] aware of any new treatments for XLH?  Please write down below.  OPEN | | |
| --- | --- | --- | --- |
|  | Yes | ____ |  |
|  | I am not aware | ○ |  |

| **Q11** | [ALL] Please indicate below to what extent do [P: you / C: the person you care for] agree with the following statements. Rate on a scale of 1 to 5 where 1=strongly disagree and 5=strongly agree.  SINGLE RESPONSE PER ROW. RANDOMISE | | |
| --- | --- | --- | --- |
|  | I believe current treatments for XLH are not meeting needs of people who have it and would be beneficial to have new treatments  [C: The person I care for believes that current treatments for XLH are not meeting needs of people who have it and would be beneficial to have new treatments] | 1 2 3 4 5 |  |
|  | I would be willing to try new treatments for XLH if recommended by my doctor  [C: The person I care for would be willing to try new treatments for XLH if recommended by his/her doctor] | 1 2 3 4 5 |  |
|  | I would like to know more about new treatments for XLH  [C: The person I care for would like to know more about new treatments for XLH] | 1 2 3 4 5 |  |
|  | I am unsure where to find information about new treatments for XLH  [C: The person I care for is unsure where to find information about new treatments for XLH] | 1 2 3 4 5 |  |
|  | I am actively searching for information about treatments for XLH  [C: The person I care for is actively searching for information about treatments for XLH] | 1 2 3 4 5 |  |
|  | I am confident that there will be new treatments for XLH in the future  [C: The person I care for is confident that there will be new treatments for XLH in the future] | 1 2 3 4 5 |  |

**Surgery experience**

| **Q12** | [ALL] How many surgical procedures relating to XLH have [P: you / C: has the person you care for] undergone?  NUMERIC | | |
| --- | --- | --- | --- |
|  | 1 |  |  |
|  | 2 |  |  |
|  | 3 |  |  |
|  | 4 |  |  |
|  | 5 or more |  |  |
|  | Can’t recall/ unsure |  |  |
|  |  |  |  |

| **Q13** | [IF CODES 1 TO 5 IN Q12] How many of these procedures were osteotomies (which involve the breaking or reshaping and resetting of leg bones)?  NUMERIC | | |
| --- | --- | --- | --- |
|  | 1 | 1 |  |
|  | 2 | 2 |  |
|  | 3 | 3 |  |
|  | 4 | 4 |  |
|  | 5 or more | 5 |  |
|  | Can’t recall/ unsure | 6 |  |
|  |  |  |  |

| **Q14** | [IF CODES 1 TO 5 IN Q13] How old [P: were you / C: was the person you care for] when the first osteotomy occurred? NUMERIC | | |
| --- | --- | --- | --- |
|  |  | ________ | years old |
|  | Can’t recall/ unsure | ○ |  |
|  |  |  |  |

| **Q14a** | [IF CODES 1 TO 5 IN Q13] If [P: you have / C: the person you care for has] had multiple osteotomies, how old were [P: you / C: the person you care for] when the last osteotomy was performed? Please enter your age below.  OPEN ENDED NUMERIC | | |
| --- | --- | --- | --- |
|  |  |  | years old |
|  |  |  |  |
|  | Can’t recall | ○ |  |

| **Q14b** | [IF CODES 1 TO 5 IN Q13] [P] Is there a possibility of having further osteotomies in the future?  [C] Is there a possibility that the person you care for will have further osteotomies in the future?  SINGLE RESPONSE | | |
| --- | --- | --- | --- |
|  | Yes | 1 |  |
|  | No | 2 |  |
|  | Unsure | ○ |  |

| **Q14c** | [IF YES CODE IN Q13] [P] When do you think you will have you next osteotomy?  [C] When does the person you care for will have his/her next osteotomy?  OPEN ENDED | | |
| --- | --- | --- | --- |
|  |  |  |  |
|  |  |  |  |
|  |  |  |  |

| **Q15** | [IF CODES 1 TO 5 IN Q12] On average, how long after osteotomy did it take before [P: you were / C: the person you care for was] able to walk unassisted?  SINGLE RESPONSE | | |
| --- | --- | --- | --- |
|  | About 1 – 2 months |  |  |
|  | About 3 -4 months |  |  |
|  | About 5 – 6 months |  |  |
|  | More than 6 months |  |  |
|  | Can’t recall/ unsure |  |  |
|  |  |  |  |

| **Q16** | [IF CODES 1 TO 5 IN Q12] [P] Now thinking about all of your XLH surgical procedures, how many involved major dental procedures, such as the removal of teeth?  [C] Now thinking about all of the XLH surgical procedures, how many involved major dental procedures, such as the removal of teeth?  SINGLE RESPONSE | | |
| --- | --- | --- | --- |
|  | 1 |  |  |
|  | 2 |  |  |
|  | 3 |  |  |
|  | 4 |  |  |
|  | 5 or more |  |  |
|  | None |  | SKIP TO Q18 |
|  | Can’t recall/ unsure |  |  |

| **Q17** | [IF CODES 1 TO 5 IN Q16] How old [P: were you / C: the person you care for] when these major dental procedures first occurred (i.e. removal of teeth)? OPEN ENDED NUMERIC | | |
| --- | --- | --- | --- |
|  |  | ________ | years old |
|  | Can’t recall/ unsure | ○ |  |

| **Q17a** | [IF CODES 1 TO 5 IN Q16] If [P: you have / C: the person you care for has] had multiple major dental procedures, such as tooth removal, how old [P: were you / C: was the person you care for] when the final tooth removal was performed? Please enter age below.  OPEN ENDED NUMERIC | | |
| --- | --- | --- | --- |
|  |  | ____ | years old |
|  |  |  |  |
|  | Can’t recall | ○ |  |

| **Q17b** | [IF CODES 1 TO 5 IN Q16] In total, how many teeth [P: have you / C: has the person you care for] had removed? Please enter number below.  OPEN ENDED NUMERIC | | |
| --- | --- | --- | --- |
|  | Baby teeth (1-20) | ____ |  |
|  | Adult teeth (1-32) | ____ |  |
|  | Can’t recall | ○ |  |

**XLH Australia**

| **Q18** | [ALL] XLH Australia plans to launch the #FingersX campaign to highlight the hopes and expectations of the XLH community. Can [P: you / C: the person you care for] please complete the following sentence?  ***When it comes to XLH, I’m hoping for*** *…*  OPEN ENDED | | |
| --- | --- | --- | --- |
|  |  |  |  |
|  |  |  |  |
|  |  |  |  |

**We value your feedback! Thank you for sharing your experiences with us.**
