# Supplementary material for: What is it like living with X-linked hypophosphatemia?: results from an Australian consumer survey
Source: JBMR Plus. 2025 Dec 6;9(Suppl 5):v3–v13. doi: 10.1093/jbmrpl/ziaf027 (PMC12723801; doi:10.1093/jbmrpl/ziaf027)
Supplement: Supplemental_Appendix_2_ziaf027 [file supplemental_appendix_2_ziaf027.docx]

**Supplemental Appendix 2: Free Text Answers to Question:**

**“When it comes to XLH, I am hoping for….”**

| I am hoping for more awareness of this rare disease, more understanding of burdens of disease, inclusion for the whole population treatment and better clinician understanding and treatment. #FingersX for the XLH community and some normality in their lives. |
| --- |
| Better treatments; better understanding of xlh among the medical profession; inclusive society |
| Crystiva. My daughters is on and it has changed her life. I really can't wait.to try it myself. My body aches and hurts all day |
| My legs to stop hurting and become straight |
| Greater awareness and better treatments |
| New treatment |
| Better access to newer treatments and for all XLH's to continue to receive treatment after we turn 18 we shouldn't have to fight for proper care. |
| PBS approval of Burosumab for adult & paediatric use. |
| Treatments/treating doctors, taking care of pain, not just physical symptoms. And acknowledging the mental burden of pain. |
| New treatments! So many other countries have access to the new burosamaub treatment as it has worked wonders for the trial participants. I am wishing we could have it available in Australia also. This would change my life |
| Burosumab to finally rid phosphate in which upsets my stomach More support No pain People to understand me including medical people |
| A world in which I feel normal |
| Affordable Access to better treatments. I hope not have to fight to get treatment for XHL. Treatment for adults is next to non even tho our disease gets worse with age. |
| More efficient/less disgusting & salty medication. Overall easier to manage medication. Burosomab & Crysvita is currently beyond the reach of those over 18 which is sad & disheartening. At 20 Life is hard, & has always been hard, with phosphate & calcitriol taken many times a day when your very symptomatic. |
| A solution that doesn't require fortnightly injections |
| A life without pain where I can fit in |
| Life long treatment that improves my ability to participate in the community. |
| Access to the latest medication |
| Better medication for children that prevents surgeries to align their legs |
| No pain in my legs and no more broken bones. |
| Any sort of treatment improvement. |
| Straight legs |
| New and accessible treatments which can help resolve my symptoms/comorbidities, allowing me to live my desired lifestyle. |
| Burosumab to be approved by the PBS for children and adults to help secure a healthy and pain-fee future. |
| The chance to grow and run as fast as my friends |
| access to new treatment for children and adults |
| availability of treatment for all |
| More awareness in the community and more alternative treatments for thos that have XLH. |
| Better treatment with less side effects |
| I hope everyone can be reached with medicare the new treatment for XLH |
| Crysvita being available to all of us, especially for our children who deserve a better quality of life than we had |
| Crysvita gets approved by the PBS as the xlh community needs it! Many other countries have approved it, Australia can't be left behind! Xlh is a serious rare disease that impact people's life and their mental health. We need to give all patients the best treatment available, which is Crysvita, to give them hope to live a normal life, like everyone deserves |
| Get my son, and all the other Australian kids and adults, on Crysvita as soon as possible, so they can have a normal life |
| To get Crysvita on the PBS asap!! We need this life changing treatment, we can't wait any longer please! |
| better understanding from medical professionals regarding pain experienced in daily life and new accessible treatments for all age groups |
| Treatment with fewer side effects which can resolve my symptoms |
| The crysvita to be approved by the public health system,so my parents (who otherwise couldn't afford it) could provided it to me |
| A cure/a treatment that helps everyone to live a healthy life without bullying. A cure/treatment that is covered by the government and it is available to everyone, no mater the age, gender, ethnicity, background and passport. |
| More accessible, compassionate care by GPs and public services. Better education and understanding of the condition and the impacts of day-to-day life. Accessibility of services and mediciations such as Burosomab at low-cost for adults and children alike. |
| Crysvita to be approved by the PBS |
| better treatment. better medication be approved and finanically cost effective for patiens |
| new medication available to all who have XLH and be cost effective financlally to those, better opportunities to be listened too |
| everyone can have a chance to be treated with crysvita |
| Crysvita being approved for everyone to get it at an acceptable price! We need this life changing treatment! |
| The new treatment crysvita to get approved asap for everyone, especially our children. There isn't nothing else we could ask for. |
| Crysvita to be paid by the government because my parents can't afford it |
