# Supplementary material for: What is it like living with X-linked hypophosphatemia?: results from an Australian consumer survey
Source: JBMR Plus. 2025 Dec 6;9(Suppl 5):v3–v13. doi: 10.1093/jbmrpl/ziaf027 (PMC12723801; doi:10.1093/jbmrpl/ziaf027)
Supplement: CHERRIES_checklist_ziaf027 [file cherries_checklist_ziaf027.docx]

| **Item Category** | **Checklist Item** | **Response** |
| --- | --- | --- |
| **Design** | Describe survey design | See manuscript text |
| **Institutional Review Board approval and informed consent process** | IRB approval | N/A |
|  | Informed consent | Informed consent was implied by completion of survey.  Participants were advised on length of survey, details of data collection and storage, the investigators and purpose of the study. |
|  | Data protection | No personal or identifiable information was collected. |
| **Development and pre-testing** | Development and testing | The survey was initiated and designed by XLH Australia executive members, and conducted by Elbow Insight and Strategy, an independent market research agency. |
| **Recruitment process and description of the sample having access to the questionnaire** | Open versus closed survey | Open |
|  | Contact mode  Advertising the survey | XLH Australia subscribers were invited to participate via social media (Facebook) and email |
| **Survey administration** | Web/E-mail | XLH Australia subscribers were invited to participate via social media (Facebook) and email |
|  | Context | Members of XLH Australia |
|  | Mandatory/voluntary | Voluntary |
|  | Incentives | No incentives offered |
|  | Time/Date | The survey was available to complete between 13^th^ December 2021 and 21^st^ January 2022 |
|  | Randomisation of items or questionnaires | Yes |
|  | Adaptive questioning | Yes |
|  | Number of items | Maximum 31 |
|  | Number of scores (pages) | Variable |
|  | Completeness check | Yes |
|  | Review Step | No |
| **Response rate** | Unique site visitor | N/A |
|  | View rate | N/A |
|  | Participation rate | Based on XLH Australia subscribers, 23% responded to this survey request. |
|  | Completion rate | 100% |
| **Preventing multiple entries from the same individual** | Cookies used  IP Check  Log file analysis  Registration | This was not done.  It is possible that a person with XLH who is also a carer for a child with XLH completed the survey twice. |
| **Analysis** | Handling of incomplete questionnaires | N/A |
|  | Questionnaires submitted with atypical timestamp | N/A |
|  | Statistical correction | N/A |

**Supplemental Appendix 3: CHERRIES checklist**
